# Identifying and prioritising midwifery care process metrics and indicators: a Delphi survey and stakeholder consensus process

**DOI:** 10.1186/s12884-019-2346-z

**Published:** 2019-06-10

**Authors:** Declan Devane, Nora Barrett, Anne Gallen, Mary Frances O’Reilly, Margaret Nadin, Gillian Conway, Linda Biesty, Valerie Smith

**Affiliations:** 1 School of Nursing and Midwifery & HRB-Trials Methodology Research Network National University Ireland Galway, Galway, Ireland; 2 School of Nursing and Midwifery, National University Ireland Galway, Galway, Ireland; 3 Nursing and Midwifery Planning and Development Unit, Health Services Executive, North-West Galway, Ireland; 4 Nursing and Midwifery Planning and Development Unit, Health Services Executive, West/Mid-West Galway, Ireland; 5 Nursing and Midwifery Planning and Development Unit, Health Services Executive, Dublin, North East Ireland; 6 School of Nursing and Midwifery, Trinity College Dublin, Dublin, Ireland

**Keywords:** Quality care metrics, Midwifery care processes, Delphi survey

## Abstract

**Background:**

Measuring care processes is an important component of any effort to improve care quality, however knowing the appropriate metrics to measure is a challenge both in Ireland and other countries. Quality of midwifery care depends on the expert knowledge of the midwife and her/his contribution to women and their babies’ safety in the healthcare environment. Therefore midwives need to be able to clearly articulate and measure what it is that they do, the dimensions of their professional practice frequently referred to as midwifery care processes. The objective of this paper is to report on the development and prioritisation of a national suite of Quality Care Metrics (QCM), and their associated indicators, for midwifery care processes in Ireland.

**Methods:**

The study involved four discrete, yet complimentary, phases; i) a systematic literature review to identify midwifery care process metrics and their associated measurement indicators; ii) a two-round, online Delphi survey of midwives to develop consensus on the set of midwifery care process metrics to be measured; iii) a two-round online Delphi survey of midwives to develop consensus on the indicators that will be used to measure prioritised metrics; and iv) a face-to-face consensus meeting with midwives to review the findings and achieve consensus on the final suite of metrics and indicators.

**Results:**

Following the consensus meeting, 18 metrics and 93 indicators were prioritised for inclusion in the suite of QCM Midwifery Metrics. These metrics span the pregnancy, birth and postpartum periods.

**Conclusion:**

The development of this suite of process metrics and indicators for midwifery care provides an opportunity for measuring the safety and quality of midwifery care in Ireland and for adapting internationally. This initial work should be followed by a rigorous evaluation of the impact of the new suite of metrics on midwifery care processes.

**Electronic supplementary material:**

The online version of this article (10.1186/s12884-019-2346-z) contains supplementary material, which is available to authorized users.

## Background

Midwives engage daily in numerous healthcare interventions where their knowledge, clinical expertise and professional judgement guide and influence their decision-making to ensure high quality, safe care delivery. Knowing however what *quality* midwifery care is, and how to measure it has always been a challenge, both in Ireland and internationally [1]. Many quality improvement approaches in maternity tend to focus on care outcomes, such as mortality and morbidity, length of hospital stay, neonatal or maternal admissions to intensive/special care and readmission rates. For example, the top ten most frequently used outcomes in 32 newly published 2011 Cochrane systematic reviews of intrapartum interventions were; admission to neonatal intensive care unit, maternal satisfaction, Apgar scores < 7 at 5 min, perinatal mortality, breastfeeding rates, caesarean section, instrumental birth, pain, adverse events and infection [2]. Measuring outcomes, which may be used to reflect the quality of care, is an important healthcare indicator. To determine however the quality of midwifery care, and in particular midwives contribution to the safety of women and their infants, requires midwives to be able to clearly articulate and measure what it is that they do; that is, midwifery clinical care processes.

Measuring care processes enables healthcare providers to have insight to the quality of care delivery and to establish improvement action-plans that will ultimately lead to better outcomes for maternity services users. In the absence of this, confirming associations between care processes and short- or long-term outcomes for pregnant/postpartum women can be challenging. This is because care processes extend beyond usual care outcomes, and hold implications for how care is provided and evaluated, as well as measured [3]. Because midwives represent the largest group of healthcare professionals in the care of women and babies [4] it is important that their work is made visible and that their significant contribution to maternity outcomes is recognised.

In 2016, the Office of Nursing and Midwifery Services Directorate in Ireland commissioned a national research study to establish the important dimensions of nursing and midwifery care processes that should be measured [5]. These dimensions aimed to reflect care delivery that is sensitive to the influences of nurses and midwives aligned to evidenced-based clinical practice guidelines and standards developed for and within the context of nursing and midwifery care in Ireland. The culmination of this work has resulted in a suite of seven *Quality Care Metrics* (QCM) reports that outline these metrics and associated indicators in the healthcare areas of Midwifery, Children’s Community/Public Health, Acute, Older People, Mental Health and Intellectual Disability [6]. The objective of this paper is to report on the development and prioritisation of a national suite of QCM, and their associated indicators, for midwifery practice in Ireland. Additional file 1 presents the midwifery work-stream working group members.

## Methods

The study comprised of four discrete, yet complimentary, phases. In phase 1 a systematic review to bring together available and relevant literature on reported quality care process metrics and associated indicators across all seven of the work-stream areas (Midwifery, Children’s Community/Public Health, Acute, Older People, Mental Health and Intellectual Disability) to inform the development of a suite of process sensitive metrics and their associated indicators. Metrics and indicators identified in the systematic review were subsequently tagged against their relevant work-stream area and used to develop work-stream specific surveys for use in phases 2 and 3. Phases 2 and 3 consisted of two by two-round e-Delphi surveys to identify and prioritise a suite of metrics (phase 2) and their indicators (phase 3) for use in measuring the quality of midwifery care processes in Ireland. An e-Delphi survey is a research method that involves a series of questionnaires, called ‘rounds’, administered electronically to a panel of relevant stakeholders on a topic under investigation so as to gather their opinions. The results of each round are presented to participants in subsequent rounds, with participants asked to provide their opinion again based on the knowledge of the collective group results from the previous round. It has been described as an optimal design for facilitating consensus-building on a topic under investigation [7]. The fourth and final phase involved a face-to-face consensus meeting with midwives (*n* = 19) to review the findings from the Delphi surveys and to agree on the final suite of QCM, and their respective indicators, for midwifery care.

### Phase 1: systematic review

#### Inclusion criteria

To be included in the review the study/report had to include;

- Participants: registered midwives or nurses working in any of the seven work-stream areas of health care services, or persons in receipt of midwifery or nursing or care from these care services;
- Exposure: midwifery or nursing quality care processes (metrics or indicators). The research team defined a *quality care process metric* as a quantifiable measure that captures quality in terms of *how* (or to what extent) midwifery or nursing care is performed in relation to an agreed standard. The research team defined a *quality care process indicator* as a quantifiable measure that captures *what* midwives or nurses are doing to provide that care in relation to a specific tool or method;
- Outcomes: a specific quality process in use or proposed for use;
- Type of study: any study design.

#### Searching and selection

The following databases were searched for relevant literature; PubMed, EMBASE, PyscINFO, ASSIA, CINAHL, the Cochrane Database of Systematic Reviews, Central Register of Controlled Trials (CENTRAL), and the Database of Abstract of Reviews of Effects (DARE). Searches were restricted to 2007–2017 to enhance temporal relevancy of retrieved records. No restrictions on study design, outcomes, controls, comparators or language were applied. The search strategy used to guide the search was “nurs*:ab,ti OR midwi*:ab,ti AND (‘minimum data set’:ab,ti OR indicator*:ab,ti OR metric*:ab,ti OR ‘quality measure*’:ab,ti) AND [english]/lim AND [2007-2017]/py.” Grey literature was obtained from both database searches and unpublished materials literature submitted by members of the work-stream working groups or from other maternity units. Citations identified from the search were screened independently by pairs of two reviewers. Any disagreements were resolved between the two reviewers, or if necessary, a third reviewer was consulted. At full text screening, included studies were tagged to the specific work-stream. Full-text studies relevant to each work-stream were subsequently reviewed by two reviewers (NB and DD for midwifery) from the appropriate work-stream.

#### Data extraction and results

**Fig. 1:**
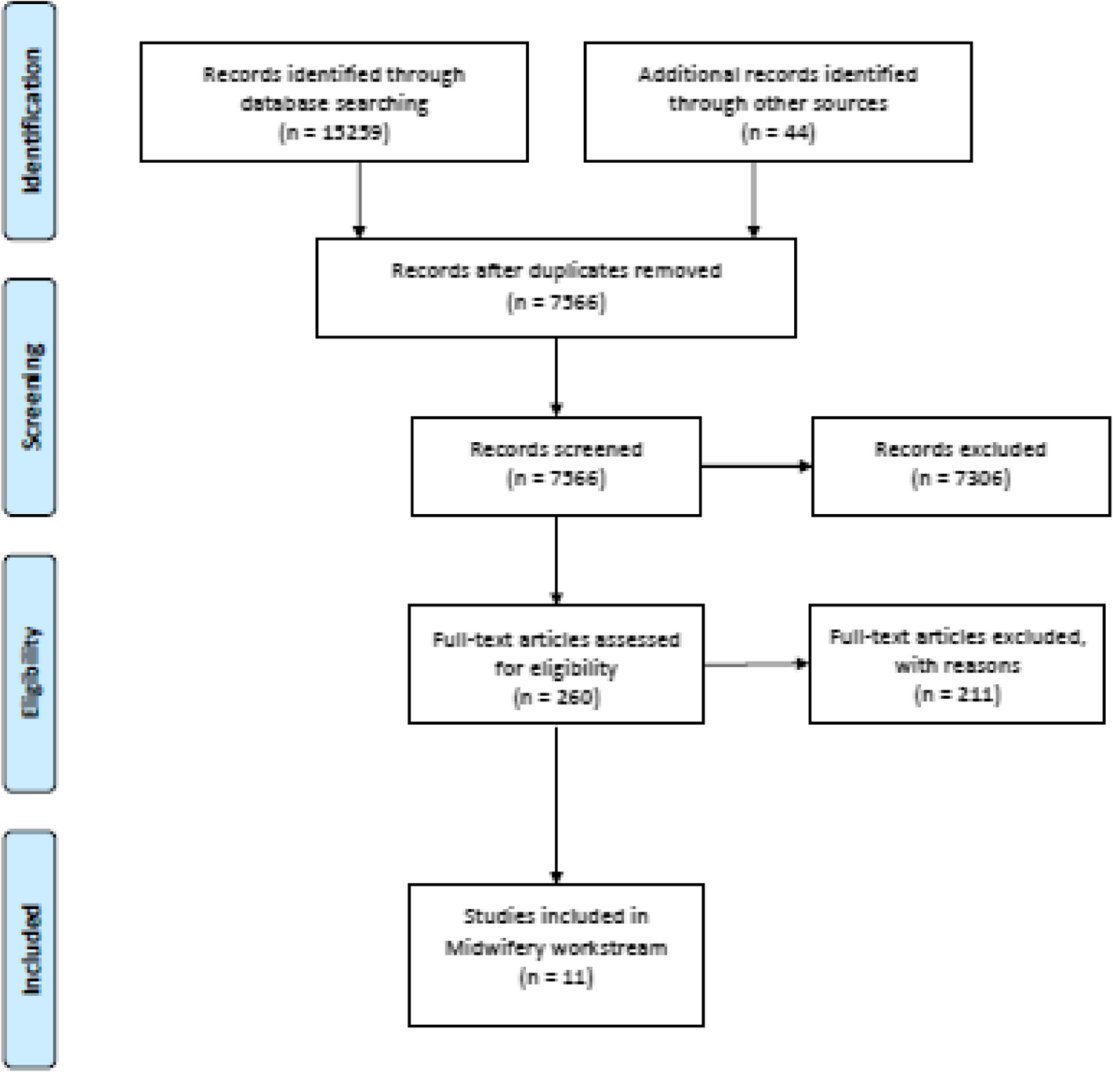
Search and selection flow diagram

In total, 7524 unique citations were identified across the seven work-streams. All citations were screened independently for inclusion by two reviewers. Following title and abstract screening, 260 were identified for full text screening after which 206 were excluded. Of the 54 remaining studies/reports, 12 were tagged as relevant to the midwifery work-stream. One of these was later excluded resulting in 11 included published papers [8–18]. An additional 42 citations were identified for the midwifery work-stream through grey literature searches. Of these 42 citations, four were excluded for not relating to midwifery or nursing quality care processes and the remaining 38 [19–57] were included as relevant. This resulted in the inclusion of 49 papers, in total contributing midwifery work-stream data (Fig. 1). Of note, the previously existing suite of midwifery care process metrics from the *Midwifery Standard Operating Procedure for Nursing and Midwifery Quality Care Metrics* [57] was identified in the grey literature search and included. These metrics are presented in Additional file 2.

Midwifery work-stream specific data extraction was conducted by two reviewers (NB & DD) using a purposefully designed data extraction tool. Data abstracted included: study aim/objective, study population, study context/setting, midwifery process in current/proposed use, measure (metric/indicator) of midwifery care process, tool or method used to measure metric, and standard/statement of defined level of quality. The review sought to identify reported quality care process metrics and associated indicators, which would later be prioritised. We did not critically appraise the reports contributing the metrics and associated indicators because we would not have used such appraisal to exclude metrics and indicators from subsequent inclusion in the prioritisation phases. The results of the systematic review identified a total of 44 metrics and 124 associated indicators. Duplicate metrics and indicators were removed. Members of the working group (see Additional file 1 for midwifery work-stream working group members) identified metrics and indicators not focussed on midwifery care processes. These metrics were reviewed and discussed by the working group. Duplicate metrics and indicators and those not focussed on midwifery care processes were removed following discussion and agreement by the working group, distilling the 44 original metrics to 22. These 22 metrics were included in the first round of the phase 2 Delphi survey instrument, which sought to prioritise the metrics.

### Phases 2 and 3: Prioritising metrics and indicators (Delphi surveys)

#### Participants and sample size

The target population for the Delphi studies was any midwife working in any sphere of midwifery practice in the Republic of Ireland. With the support of The Office of the Nursing and Midwifery Services Directorate (ONMSD), Senior Clinical Managers distributed an information pack to potential participants within their respective hospital or community healthcare area. Potential participants were identified by managers by sending information on how to participate to all staff within each respective area for which managers had responsibility. This information pack provided potential participants with information on the study, invited participation and asked those who wished to participate to complete a short form containing their contact details, including their email address, and to return this form to the Senior Clinical Manager. The managers and any potential participants could also contact the research team directly to clarify any issues or seek further information about the study prior to making a decision to participate. Snowball sampling was used also, whereby participants were asked to forward the invitation to others whom they regarded as meeting the sampling criteria. Two email invitations were sent to all potential participants, 1 week apart. There is an absence of guidance on optimal sample size requirements for consensus development studies such as this. We therefore estimated our required completed survey sample sizes based on that which would be required for the sample to be representative of a given total population of 1884 midwives practicing in Ireland using a 95% confidence level and a confidence interval of ±5. Estimates indicated we required 318 completed surveys.

#### Metric Delphi study

Phase 2 involved a two-round Delphi to prioritise the metrics. In the first-round, the instrument contained a short questionnaire seeking participant demographic data and the metrics rating instrument, which contained the 22 metrics identified in the systematic review. To facilitate the capture of metrics not identified in the systematic review, participants were invited, in this round, to add any further ‘new’ metrics that they considered important or relevant for inclusion in the metric dataset. Participants were asked to rate the importance of these metrics for inclusion using a 9-point Likert scale (1–3 = not important, 4–6 = unsure of importance and 7–9 = important), based on the 9-point Delphi scale, with a 70% cut-off of participants having to rate the metric as ‘important’ used for developing core outcome sets in healthcare (http://www.comet-initiative.org/). In round 2, participants who responded to round 1 were presented again with all of the metrics after analysis of responses from round 1 (see ‘Data analyses’ below for details). Additional metrics identified by participants in round 1 were included in round 2. For each metric retained from round 1, the overall rating results (percentages) for each metric was presented. Participants were also sent confidential copies of their individual Round 1 survey responses and asked to re-rate the importance of each metric with knowledge of their own and the overall group’s previous rating for that metric. In addition, participants were asked to rate the metrics identified newly from round 1. All ratings used the same Likert-type scale used in round 1.

#### Indicator Delphi study

Phase 3 involved a two-round Delphi to prioritise the indicators. The round 1 indicator instrument contained a short questionnaire seeking participant demographic data and the rating instrument containing metrics identified in phase 2 and the indicators for these metrics identified from the systematic review. To facilitate the capture of indicators not identified in the review, participants were invited to add any further ‘new’ indicators they considered important or relevant for inclusion as an indicator to measure the respective metric(s). The same rating scale used in phase 2 was used for phase 3, whereby participants were asked to rate the importance of these indicators for inclusion in the respective metric on a 9-point Likert scale (1–3 = not important, 4–6 = unsure of importance and 7–9 = important). Participants who responded to round 1 were presented in round 2 with all of the metrics and their indicators after analysis of responses from round 1 (see ‘Data analyses’ below for details). Additional indicators identified by participants in round 1 were included in round 2. For each indicator retained from round 1, the rating results (percentages) for each were presented. Participants were sent their individual round 1 survey responses and asked to re-rate the importance of each indicator with knowledge of their and the overall participant’s previous rating for that indicator. In addition, participants were asked to rate indicators identified newly from round 1. The same Likert-type scale (i.e. 1–9 scale ranging from not important to important) used in round 1 was used in round 2.

#### Data analyses

Consensus on inclusion of a metric, following phase 2, round 2, was determined where 70% or more participants rated the metric as 7 to 9 and less than 15% of participants rated the metric as 1 to 3. Similarly, in phase 3, round 2, consensus on inclusion of an indicator was determined where 70% or more participants rated the indicator a 7 to 9 and less than 15% of participants rated the indicator as 1 to 3.

#### Ethics

Participation in the study was voluntary. All potential participants received a study information sheet, which outlined the purpose of the study, the risks and benefits of participation, and time commitment and were afforded the opportunity to ask any questions including at the end of each Delphi round. All participants had to indicate their explicit consent to participate by clicking on an *‘I agree’* button at the end of the online participant information sheet before they could access the survey. In phase 4, potential consensus meeting participants were given a participant information leaflet containing the necessary information on which they could base their decision on participating, or not, in the consensus meeting. Written consent to participate was then obtained from each participant at the meeting. Ethical approval to conduct this study was granted by the Research Ethics Committee, National University of Ireland Galway.

## Results

A total of 441 midwives expressed an interest, by email, in participating in phase 2 (prioritising metrics), of which 263 participated in round 1 of the metric survey. Just over one third of participants were staff midwives (34.6%, *n* = 91) and almost one quarter were clinical midwife managers (grade 2) (24.3%, *n* = 64). A large proportion of participants identified their grade of midwifery as ‘other’ (12.2%, *n* = 32). Of these, most were either clinical skills facilitators (18.8%, *n* = 6) or clinical placement coordinators (18.8%, *n* = 6) (Table 1). Of the 263 respondents who completed round 1, 69.1% (*n* = 183) completed Round 2. Over half of the respondents to round 2 were either staff midwives (26.8%, *n* = 49) or clinical midwife managers (grade 2) (29.5%, *n* = 54). A large proportion of the participants in round 2 also identified their grade of midwifery as ‘other’, that is, clinical skills facilitators or clinical placement coordinators (27.5%, *n* = 38) (Table 1).

**Table 1:** Midwifery participants by grade: Phase 2 metric survey

Grade of Midwife	Round 1 Total = 263 *n* (%)	Round 2 Total = 183 *n* (%)
Staff Midwife	91 (34.6)	49 (26.8)
Clinical midwife manager (1)	18 (6.8)	8 (4.4)
Clinical midwife manager (2)	64 (24.3)	54 (29.5)
Clinical midwife manager (3)	14 (5.3)	8 (4.4)
Assistant Director of Midwifery	20 (7.6)	17 (9.3)
Director of Midwifery	8 (3.0)	9 (4.9)
Clinical Midwife Specialist	13 (4.9)	0 (0)
Advanced Midwife Practitioner	3 (1.1)	0 (0)
Other^a^	32 (12.2)	38 (27.5)

^a^e.g. clinical skills facilitators, clinical placement coordinators

Participants rated 21 of the 22 metrics included in phase 2, round 1, as important for inclusion in the suite. In addition, nine metrics were identified newly by participants. These, plus the 21 metrics, were carried forward to round 2. In round 2, participants rated all 30 metrics as important for inclusion in the suite. These 30 metrics were discussed in detail by the midwifery working group where three (Women’s Experience, Irish-Maternity Early Warning Score (I-MEWS) and Invasive Medical Devices) were identified as having a separate process either underway or planned for which indicators were or would be developed. For this reason, these three metrics were not included in phase 3 of the project within which indicators to measure adherence to the metrics were prioritised. In addition, eight metrics were judged to overlap with other metrics and were removed. The remaining 19 metrics were carried forward to phase 3 and later to the face to face consensus meeting (phase 4) along with participants’ suggestions for where metrics may overlap.

A total of 217 midwives participated in the phase 3, round 1 Delphi survey (prioritising indicators). Just over half of the respondents were staff midwives (30.0%, *n* = 65) and clinical midwife managers (grade 2) (25.4%, *n* = 55) (Table 3). Of the 217 midwives who completed round 1, 69.6% (*n* = 151) of these completed round 2. Just over one quarter of respondents to this final round were staff midwives (25.89%, *n* = 39) and one-fifth were clinical midwife managers (grade 2) (19.9%, *n* = 30). A large proportion of participants (19.2%, *n* = 29) identified their grade of midwifery as ‘other’, which consisted largely of clinical placement coordinators (20.7%, *n* = 6) (Table 2).

**Table 2:** Midwifery participants by grade: Phase 3 indicator survey

Grade of Midwifery	Round 1 Total = 217 *n* (%)	Round 2 Total = 151 *n* (%)
Staff Midwife	65 (30.0)	39 (25.8)
Clinical Manager (1)	13 (6.0)	13 (8.6)
Clinical Manager (2)	55 (25.4)	30 (19.9)
Clinical Manager (3)	15 (6.9)	6 (4.0)
Assistant Director of Midwifery	17 (7.8)	15 (9.9)
Director of Midwifery	12 (5.5)	8 (5.3)
Clinical Midwife Specialist	6 (2.7)	8 (5.3)
Advanced Midwife Practitioner	3 (1.4)	3 (2.0)
Other^a^	31 (14.3)	29 (19.2)

^a^e.g. clinical skills facilitators, clinical placement coordinators

Of the 109 indicators included in phase 3, participants, in round 1, rated all as important for inclusion in the midwifery metrics suite. In addition, 1 indicator was newly identified by participants. In round 2, participants rated the 110 indicators as important for inclusion in the suite. Following detailed review and discussion by the working group, seven indicators were judged to lack clarity, were potentially ambiguous and were removed. The remaining 103 indicators and the associated 19 metrics, were carried forward to the face to face consensus meeting.

### Phase 4 consensus meeting

A face-to-face meeting with key stakeholders (midwives) was held in Nov 2017 to review the findings from the Delphi surveys and build consensus on the metrics and respective indicators. In total, 19 midwives participated in the face to face consensus meeting. Each of the 19 maternity units in Ireland had a midwifery representative and participants represented all grades of midwives.

At the consensus meeting, participants were provided with paper copies of the list of 19 metrics and 103 indicators resulting from the Delphi surveys as well as the percentage rating for each metrics and indicator. Participants were also provided with a Judgement Framework Tool (Table 3), adapted from Flenady et al. [58] to guide participants in judging if the metric/indicator was appropriate for inclusion in the final suite of metrics.

**Table 3:** QCM Judgement Framework Tool^a^

Domain	Description
Process Focused	The metrics/indicator contributes clearly to the measurement of nursing or midwifery care processes.
Important	The data generated by the metric/indicator will likely make an important contribution to improving nursing or midwifery care processes.
Operational	Reference standards are developed for each *metric* or it is feasible to do so. The *indicators* for the respective metric can be measured.
Feasible	It is feasible to collect and report data for the metric/indicator in the relevant setting.

^a^Adapted from Flenady et al. [54]

Participants of the consensus meeting voted YES or NO on whether they felt that each metric and indicator should be included in the final suite using an anonymous electronic voting system. To be included in the final suite, a metric or indicator required a YES vote by 70% (*n* = 13) or more participants. At the conclusion of the consensus meeting, agreement on 18 metrics with 93 associated indicators for inclusion in the final suite of Midwifery Quality Care Metrics was achieved (Table 4).

**Table 4:** Agreed Metrics and Indicators Following Midwifery Consensus Meeting

Metric (*n* = 18)		Indicators (*n* = 93)
Midwifery Plan of Care	1	A midwife’s plan of care is evident and reflects the woman’s current condition including referral where appropriate
	2	Appropriate midwifery care based on the assessment and plan is reordered
Booking	1	The woman’s name and healthcare record number are on each page/screen
	2	All previous pregnancies and outcomes are recorded
	3	Past medical/surgical/family/genetic/social/medication (as appropriate) histories are recorded
	4	The allergy status is recorded
	5	Infection status /alert is recorded
	6	The blood pressure, and gestation at booking is recorded
	7	There is evidence of assessment of antenatal risk factors recorded
	8	Whether a blood transfusion is acceptable to the woman is recorded
	9	There is evidence of assessment for mental health illnesses recorded
	10	There is evidence of routine inquiry for domestic violence recorded
	11	There is evidence that infant feeding has been discussed with the woman and recorded
	12	There is evidence that health information relating to pregnancy has been given and recorded
Abdominal examination (after 24 weeks gestation) on current or last assessment	1	Abdominal inspection findings are recorded
	2	Palpation-Fundal height in cms (where appropriate) is recorded
	3	Palpation-Lie is recorded
	4	Palpation-Presentation (where appropriate) is recorded
	5	Palpation-Position (where appropriate) is recorded
	6	Palpation-Engagement (where appropriate) is recorded
	7	Palpation-Fetal activity (if present) is recorded
	8	Auscultation-Fetal heart rates-Use of Pinard or hand held Doppler with a record of fetal heart rate in beats per minute (BPM)
Intrapartum fetal Wellbeing	1	There is recorded evidence of fetal heart monitoring with Pinard/Doppler on initial assessment
	2	When using intermittent auscultation, the fetal heart is recorded at least every 15 min in the 1st stage of labour and at least every 5 min in the 2nd stage of labour
	3	There is recorded evidence of date and time of infant’s birth in the labour record
	4	Colour and volume of liquor are recorded
Intrapartum fetal wellbeing cardiotocography (CTG)	1	There is recorded evidence of indication for cardiotocography (CTG)
	2	The date/time is validated and recorded at the start of CTG
	3	The woman’s name and hospital number are recorded on the CTG by the midwife
	4	The maternal pulse is recorded on the CTG strip on commencement of the CTG tracing
	5	There is recorded evidence of systematic CTG interpretation occurring hourly (baseline, variability, accelerations, decelerations, uterine activity and plan of care)
	6	There is recorded evidence that CTGs of concern have been reviewed by the senior midwife and/or obstetrician
Intrapartum Maternal wellbeing	1	There is recorded evidence of recording of maternal vital signs during labour according to the woman’s condition
	2	A narrative is recorded at least hourly, to provide a record of the woman’s condition
	3	Indication for vaginal examination is recorded
	4	Consent to perform vaginal examination is recorded
	5	There is recorded evidence of abdominal examination prior to vaginal examination.
	6	There is evidence of systematic record keeping of the findings of all vaginal examinations
	7	There is recorded evidence that a discussion has occurred with the woman about her care to include birth preferences
	8	There is recorded evidence of contraction assessment at least every 30 min
	9	There is recorded evidence of date and time of onset of each stage of labour
	10	The name and designation of the person professionally requested to review the woman is recorded (as appropriate)
	11	Indication for amniotomy is recorded
	12	Consent for amniotomy is recorded
	13	Indication for administration of oxytocin is recorded
	14	Consent for administration of oxytocin is recorded
	15	There is recorded evidence that oxytocin infusion has been reduced or stopped when uterine tachystystole is present
	16	Where a CTG is of concern, there is recorded evidence that the oxytocin infusion was reduced or discontinued and a medical review was undertaken
	17	There is recorded evidence of findings of assessment for perineal trauma
	18	Where perineal repair is necessary and is performed by midwife, there is recorded evidence of repair
	19	There is recorded evidence of estimated blood loss at birth
	20	The date, time and method of birth are recorded
Risk assessment for venous thromboembolism (VTE) in pregnancy and the puerperium	1	There is recorded evidence of venous thromboembolism (VTE) assessment on admission
	2	There is recorded evidence of VTE assessment postnatally
Immediate post birth care	1	Maternal vital signs are recorded on the IMEWS chart, prior to transfer to the postnatal ward
	2	Maternal urinary output is recorded
	3	Skin to skin contact is recorded
	4	Breast feeding initiation time is recorded for a woman who chooses to breastfeed
	5	Neonatal condition at birth (live, neonatal death, fetal death) is recorded
	6	Findings of initial systematic examination of the newborn is recorded
Communication (Clinical Midwifery Handover)	1	Mother- Identification of risk factors in handover is recorded
	2	Baby- Confirmation of identify band checking is recorded
	3	Baby- Gender of newborn is recorded
	4	Baby- Security tag is recorded as present and active
Pain management (other than labour)	1	Woman’s response to actions taken to reduce pain are recorded
Infant feeding	1	Method of infant feeding is recorded
	2	Assessment of effectiveness of baby feeding is recorded
	3	The actions taken if feeding is ineffective are recorded
Postnatal care (daily midwifery care processes)	1	There is recorded evidence of ongoing postnatal education being offered to the woman
	2	There is recorded evidence of daily assessment of the mother (as per national health care record/local policy)
	3	There is recorded evidence of how well the woman is coping postnatally
	4	There is recorded evidence of daily assessment of the neonate (as per national health care record/local policy)
Post birth discharge planning for home	1	Discharge date and time are recorded
	2	The name of midwife completing discharge is recorded
	3	The destination of the woman is recorded on discharge
	4	Referral for professional skilled services (e.g. lactation consultant, physio, social work, speciality clinic, if required) is recorded
	5	There is recorded evidence of neonatal pulse oximetry screening having been performed (if appropriate)
	6	There is recorded evidence of discharge advice/discussion on health and wellbeing of self and baby
Medication administration	1	The allergy status is clearly identifiable on the front page of prescription chart.
	2	All prescribed medication is administered in accordance with local and national policies, procedures, protocols and guidelines (PPPGs)
Medication, Storage and Custody (excluding MDAs)	1	A registered midwife is in possession of the keys for medicinal product storage
	2	All medicinal products are stored in a locked cupboard or locked room
MDA Drugs	1	MDA drugs are checked & signed at each changeover of shifts by midwifery staff
	2	Two signatures are entered in the MDA drug register for each administration of an MDA drug
	3	The MDA drug cupboard is locked and keys for MDA cupboard are held by designated midwife
	4	MDA drug keys are kept separate from other medication keys
Intravenous fluid therapy	1	Fluid balance charts are completed accurately and totalled
Clinical Record Keeping	1	All entries are dated and timed (using 24 h clock)
	2	All written records are legible, in permanent ink and signed
	3	All entries are in chronological order
	4	All abbreviations/grading systems are from a national or local approved list/system
	5	Alterations/corrections are as per HSE standards and recommended practices for healthcare records management
	6	Recorded care provided by midwifery students is countersigned by a registered midwife

For the additional three metrics identified in phase 2 (i.e. Women’s Experience, Irish-Maternity Early Warning Score (I-MEWS) and Invasive Medical Devices) not forwarded to phase 3 because they were identified as having a separate process either underway or planned for which indicators were and would be developed, these indicators will be taken from the following when complete: i) Women’s Experience to be measured with HIQA/HSE National Women’s Experience Survey, ii) Invasive Medical Devices to be recorded as part of the Peripheral lines and urinary catheters care bundles, and iii) IMEWS/Observation to be recorded in the new IMEWS Guideline Audit Tool.

## Discussion

This study describes a strategy of identifying and prioritising a suite of 18 metrics and 93 associated indicators to measure midwifery care processes. Measuring the quality of the process of midwifery care is complex [59]. The metrics and indicators presented here offer an important understanding of the interplay between care delivery, measurement and care outcomes and how maternity system improvement through the actions and interventions of midwives might be achieved. The metrics and indicators are not designed necessarily to offer an exhaustive list, nor do we consider that they should be used solely in isolation of contextual issues, including variation in national/regional models of care. Organisations should aim to achieve consensus on a set of measures including structural, process and outcome data to guide the delivery of high quality safe care provision across the maternity care continuum, from antenatal through to the postpartum period. The current set of QCM and indicators were developed specifically with the Irish maternity care system in mind, and we accept that care systems can vary internationally, as well as regionally. In Ireland, for example, a national survey in 2014 indicated that 69% of 2820 surveyed women would like a model of midwifery care (e.g. midwifery-led care in hospital, home birth, birth centres) available to them, however, only 20% were able to avail of this type of care [60]. This is largely reflective of the type of maternity care offered in Ireland, with 19 maternity hospitals across the country, and the availability of only two midwifery-led units alongside consultant-led hospital units, 14 self-employed community midwives, and no stand-alone birth centres. Acknowledging this, we believe the QCM reported here can be used or adapted for use in other countries and settings, while recognising that care processes might be context specific [59].

This research process and final set of midwifery QCM and indicators were identified and prioritised using a methodologically robust and rigorous process. Importantly, the widespread engagement in the project by midwives of all grades and geographical areas nationally, via the work stream groups and project officers, has ensured that there is a real sense of ownership of the metrics and indicators from midwives across settings. This, in turn, has ensured relevance and will enhance direct transferability to clinical midwifery practice. We recognise, however, some limitations to this work. For example, our sample size falls short of the a priori sample size of 318 (using a 95% confidence level and a confidence interval of ±5); however, our sample size at round 2 of both metrics and indicators surveys achieved a 95% confidence level with confidence intervals of ±7 and ± 8 respectively. Staff midwife grades were under represented somewhat in the Delphi surveys despite extensive efforts to hear their views. While a maternity service user was a member of the project Steering Group (SG), we also acknowledge that the voice of pregnant and postpartum women and their families is largely absent. This decision was made at SG level, because the project SG felt the focus should be on midwives.

Although not the aim of our work, the systematic review in phase 1 of the study identified a dearth of evidence on studies evaluating the effectiveness of metrics and indicators on quality of care and thus, a need for research assessing such effectiveness is recommended. Follow-up on this initial work is intended via a rigorous evaluation of the impact of the new suite of metrics on midwifery care processes. Designs that control, insofar as is possible for confounding variables such as interrupted time series designs will be considered, and determined prior to implementation so that opportunities for baseline assessments are not lost.

## Conclusion

Knowing what midwives do, and how they do it, is a fundamental component to achieving high quality maternity care. The result of this study (i.e. the suite of metrics and indicators) offers a basis for embedding the concept of measurement for improvement in midwifery practice in order to assure the delivery of high quality, safe maternity care. Use of the suite of QCM will also facilitate measurement of and accountability in care provision, and will assist, ultimately, in achieving the goal of improved maternal, fetal and neonatal outcomes.

## Additional files

Additional file 1: Midwifery work-stream working group members. (DOCX 13 kb)

Additional file 2: Existing Midwifery Metrics at the Start of Quality Care Metrics Process. (DOCX 18 kb)

## Data Availability

Via request from the corresponding author.

## Abbreviations

HIQA: Health Information Quality Authority
HSE: Health Service Executive
MDA: Misuse Drugs Act
NMBI: Nursing and Midwifery Board of Ireland
NMPDU: Nursing and Midwifery Planning and Development Units
ONMSD: Office of the Nursing and Midwifery Services Director
QCM: Quality Care Metrics
SG: Steering Group
SOP: Standard Operating Procedure
