# Supplementary material for: Identifying and prioritising midwifery care process metrics and indicators: a Delphi survey and stakeholder consensus process
Source: BMC Pregnancy Childbirth. 2019 Jun 10;19:198. doi: 10.1186/s12884-019-2346-z (PMC6558705; doi:10.1186/s12884-019-2346-z)
Supplement: Supplementary file 1 — Midwifery work-stream working group members. (DOCX 13 kb) [file 12884_2019_2346_MOESM1_ESM.docx]

**Additional File 1: Midwifery work-stream working group members**

| - Office of the Nursing & Midwifery Services Director (n=1) - National Lead, Nursing and Midwifery Planning Development, Health Services Executive, North-West (n=1) - Members, Nursing and Midwifery Planning Development, Health Services Executive, West/Mid-West (n=2), Dublin North-East (n=1) - Lead Academics, National University of Ireland Galway (n=2), Trinity College Dublin (n=1) - Research Assistant, National University of Ireland Galway (n=1) - National Service User Representatives (including Directors of Midwifery, midwives in practice development roles, and staff midwives) (n=43) |
| --- |
