# Supplementary material for: Identifying and prioritising midwifery care process metrics and indicators: a Delphi survey and stakeholder consensus process
Source: BMC Pregnancy Childbirth. 2019 Jun 10;19:198. doi: 10.1186/s12884-019-2346-z (PMC6558705; doi:10.1186/s12884-019-2346-z)
Supplement: Supplementary file 2 — Existing Midwifery Metrics at the Start of Quality Care Metrics Process. (DOCX 18 kb) [file 12884_2019_2346_MOESM2_ESM.docx]

**Additional File 2: Existing Midwifery Metrics at the Start of Quality Care Metrics Process**

| **Metric** | **Indicators** |
| --- | --- |
| Medication Storage and Custody | A registered midwife is in possession of the keys for Medicinal Product Storage  All Medicinal products are stored in a locked cupboard or locked room.  All medication trolleys are locked and secured as per local organisational policy and open shelves on the medication trolley are free of medicinal products when not in use.  A Drug Formulary is available on all Med Trolleys. |
| MDA Drugs | MDA drugs are checked & signed at each changeover of shifts by midwifery staff. (By member of Day Staff & Night Staff).  Two signatures are entered in the MDA Drug Register for each administration of an MDA drug.  The MDA Drug cupboard is locked and keys for MDA cupboard are held by designated Midwife.  MDA drug keys are kept separate from other medication keys. |
| Medication Administration | The Individual’s prescription documentation provides details of individual’s legible name and health care record number.  The Individuals’ identification band has correct and legible name and healthcare record number or photo ID is in use.  The Allergy Status is clearly identifiable on the front page of the prescription chart.  Prescribed Medication not administered have an omission code entered.  The individuals’ locker and bedside/ or surrounding environment are free of unsecured prescribed medicinal products. |
| Medication Prescription | The Generic name is used for each drug prescribed  The Start date is recorded  The Prescription is written in capital letters  The correct legible Dose of the drug is recorded and not abbreviated  The Route and/or Site of Administration is recorded  The Frequency of Administration is recorded & correct timings indicated  The minimum dose interval and/or 24-hour maximum dose is specified for all “as required” or PRN drugs  The Prescription has a legible Prescriber’s Signature (in ink)  Discontinued drugs are crossed off, dated and signed by prescriber. |
| Midwifery Plan of Care: Personal Details | The Individuals Name and Healthcare Record Number are on each page/screen.  Reason for admission/attendance is recorded and the admission date and time are Recorded.  All previous pregnancies and out comes are documented.  Past medical/surgical history are recorded  The Allergy Status is clearly identifiable on relevant nursing documentation.  Infection Status /Alert is recorded.  There is evidence that the booking bloods results are recorded.  There is evidence that infant feeding has been discussed with the woman.  There is evidence that health information relating to pregnancy has been given. |
| Midwifery Plan of Care | A Midwife’s plan of Care is evident and reflects the individuals’ current condition.  All risk assessments have been completed within the set time frames as per local policy.  When a woman is considered high risk, there is documented evidence that she is referred to the appropriate medical team/obstetric team/service.  Midwives Interventions are individualised, dated, timed and signed.  Timely Evaluation of the Midwife’s plan of care is evident and has been updated accordingly. |
| NMBI Guidance | All entries are dated and timed (24-hour clock).  All written records are legible, in permanent ink and signed.  All entries are in chronological order.  All abbreviations/grading systems are from a national or local approved list/system.  Alterations/corrections are as per NMBI Guidance.  Student midwives’ entries are countersigned by the supervising midwife. |
| Monitoring in Labour | Indication and Consent to perform vaginal examinations are recorded.  Indication and consent for type of fetal monitoring is documented  There is evidence of fetal heart monitoring with Pinard/doptone/doppler on initial Assessment.  Birthplans are dated and timed and signed by attending midwife.  The name and designation of the person professionally requested to review the woman is documented.  A narrative is recorded at least hourly, to provide a record of the woman’s condition. |
| Partogram Monitoring | The partogram was commenced on diagnosis of labour onset.  Maternal blood pressure, pulse and temperature are recorded on the partogram.  The fetal heart is recorded every 15 minutes in the first stage of labour and every 5 minutes in the 2nd stage.  The frequency of uterine activity was recorded for 10 minutes at least every 30 minutes up to time of birth. |
| CTG Monitoring | The date/time is validated at the start of the CTG.  The woman’s name and hospital number are recorded on the CTG strip by the midwife.  The maternal pulse is recorded on the CTG strip on commencement of the procedure.  There is documented evidence that a pathological CTG pattern was reviewed by the Senior midwife and Registrar.  The date, time and method of birth are recorded at the end of the trace and the CTG is stored securely either electronically or at the back of chart securely. |
| Oxytocin Monitoring | Indication and consent for use of oxytocin is recorded.  Oxytocin infusion has been reduced when contraction frequency has exceeded 5 in 10 Minutes.  For a pathological CTG there is evidence that the oxytocin infusion was discontinued and a medical review was undertaken. |
| Midwifery Plan of Care: Post Delivery | Maternal observations, temperature, pulse, BP, respirations were recorded on the Early Warning Chart, prior to transfer to the postnatal ward.  Uterine involution, blood loss, condition of perineum and urinary output are documented.  Skin to skin contact is recorded.  Infant temperature is recorded.  Breast-feeding initiation time is recorded for a woman who chooses to breastfeed. |
| IMEWS/Observation | The woman’s name, date of birth and healthcare record number are on both sides of the observation chart.  Observations are dated, timed and signed.  The booking blood pressure and gestation at booking is recorded.  The IMEWS are recorded using the 24 hour clock for each entry.  In each entry, Respiratory Rate, Temperature, Maternal heart rate, systolic and diastolic blood pressure, SPO2, urinalysis, pain score and AVPU are recorded.  In each entry, the IMEWS is completed and totalled correctly  There is evidence that the care was escalated to the appropriate level as per escalation protocol (Team/On Call SHO/Registrar/Consultant as appropriate).  There is evidence of an increase in the frequency of monitoring and recording of vital signs in response to the detection of abnormal physiology.  24hr cumulative balances are evident on all fluid balance charts. |
| Invasive Medical Devices | An assessment of the insertion site is recorded daily on care plan.  The Clinical Indication for insertion of the indwelling urinary catheter is recorded. |
| Discharge Planning | There is evidence that the woman has been given information on reasons for presenting to hospital outside of appointment times.  There is documented evidence that discharge advise has been discussed with the Woman.  A Predicted Date of Discharge is documented. |
| Women’s Experience  Quality Care-Metric | 1. Are you satisfied with the cleanliness of the ward?  2. Have you observed the midwives perform hand hygiene?  3. Have you received adequate information from midwives about your medication?  4. Have midwives given you enough privacy when being examined or treated?  5. Do midwives treat you with respect and dignity on this ward?  6. Do you feel your pain has been managed appropriately  7. When using the Midwife Call bell/buzzer, is it answered within the appropriate timeframe?  8. Have midwives on the ward talked to you about going home?  9. Would you recommend this hospital/service to your family or friends? |
